# Safety and efficacy of left atrial ablation after percutaneous left atrial appendage occlusion in high-bleeding-risk patients with atrial fibrillation: a multicentre analysis from the PROCEED-AF ablation registry

**DOI:** 10.1093/europace/euag277

**Published:** 2026-09-25

**Authors:** Rachel M Kaplan, Jeanne du Fay de Lavallaz, Christian-Hendrik Heeger, Hakan Oral, Mason Yoder, David Schaack, Julian K Chun, Boris Schmidt, Jeffrey Winterfield, William H Sauer, Fernando Moreno, Jonathan W Waks, Antonio Dello Russo, Paolo Compagnucci, Pipin Kojodjojo, Ghanshyam Shantha, Laurent Roten, Tobias Reichlin, Thomas Kueffer, Kenneth Brownell, Smit C Vasaiwala, Timothy M Markman, Patrick Badertscher, Michael Kühne, Christian Sticherling, Nishant Verma, Graham Peigh, Bradley P Knight, Tom De Potter, Konstantia Paraskevi Gkini, Dhiraj Gupta, Mark T Mills, Serge Boveda, Robin Richard-Vitton, Jorge Romero, Fengxiang Zhang, Raktham Mekritthikrai, Henry D Huang

**Affiliations:** Department of Cardiology, Medical University of South Carolina, 30 Courtenay Dr, Charleston, SC 29414, USA; Department of Cardiology, University of Michigan, Ann Arbor, MI, USA; Department of Cardiology, University of Luebeck, Luebeck, Germany; Department of Cardiology, University of Michigan, Ann Arbor, MI, USA; Department of Cardiology, University of Michigan, Ann Arbor, MI, USA; Department of Cardiology, Cardioangiologisches Centrum Bethanien Frankfurt, Frankfurt, Germany; Department of Cardiology, Cardioangiologisches Centrum Bethanien Frankfurt, Frankfurt, Germany; Department of Cardiology, Cardioangiologisches Centrum Bethanien Frankfurt, Frankfurt, Germany; Department of Cardiology, Medical University of South Carolina, 30 Courtenay Dr, Charleston, SC 29414, USA; Department of Cardiology, Brigham and Women’s Hospital, Boston, MA, USA; Department of Cardiology, Brigham and Women’s Hospital, Boston, MA, USA; Department of Cardiology, Beth Israel Deaconess Medical Center, Boston, MA, USA; Department of Cardiology, University Hospital Ospedali Riuniti Umberto I-Lancisi-Salesi, Ancona, Italy; Department of Cardiology, University Hospital Ospedali Riuniti Umberto I-Lancisi-Salesi, Ancona, Italy; Department of Cardiology, Asian Heart and Vascular Center, Singapore, Singapore; Department of Cardiology, Wake Forest University Health Sciences, Winston-Salem, NC, USA; Department of Cardiology, Department of Cardiology, Inselspital, Bern University Hospital, University of Bern, Switzerland; Department of Cardiology, Department of Cardiology, Inselspital, Bern University Hospital, University of Bern, Switzerland; Department of Cardiology, Department of Cardiology, Inselspital, Bern University Hospital, University of Bern, Switzerland; Department of Cardiology, Loyola University Medical Center, IL, USA; Department of Cardiology, Loyola University Medical Center, IL, USA; Department of Cardiology, University of Pennsylvania, PA, USA; Department of Cardiology, University Hospital Basel, Basel, Switzerland; Department of Cardiology, University Hospital Basel, Basel, Switzerland; Department of Cardiology, University Hospital Basel, Basel, Switzerland; Department of Cardiology, Northwestern University, Chicago, IL, USA; Department of Cardiology, Northwestern University, Chicago, IL, USA; Department of Cardiology, Northwestern University, Chicago, IL, USA; Department of Cardiology, OLV Hospital, Aalst, Belgium; Department of Cardiology, OLV Hospital, Aalst, Belgium; Department of Cardiology, Northern General Hospital, Sheffield Teaching Hospitals NHS Foundation Trust, Sheffield, UK; Department of Cardiology, Northern General Hospital, Sheffield Teaching Hospitals NHS Foundation Trust, Sheffield, UK; Department of Cardiology, Clinique Pasteur, Toulouse, France; Department of Cardiology, Clinique Pasteur, Toulouse, France; Department of Cardiology, State University of New York Upstate Medical University, New York, USA; Department of Cardiology, Nanjing Medical University, Nanjing, China; Department of Cardiology, Rush University Medical Center, Chicago, IL, USA; Department of Cardiology, University of Chicago, Chicago, IL, USA

**Keywords:** Atrial fibrillation, Catheter ablation, Left atrial appendage occlusion, Peri-device leak

## Abstract

**Aims:**

Patients with left atrial appendage (LAAO) devices are increasingly referred for atrial fibrillation (AF) ablation, yet anticoagulation strategies remain uncertain. The goal of this study was to evaluate the safety and efficacy of catheter ablation for AF in patients with pre-existing LAAO devices and to describe patterns in anticoagulation management after ablation in this unique population.

**Methods and results:**

We collected data from 14 centres worldwide on patients with prior LAAO devices undergoing AF ablation. Each site provided clinical characteristics, procedural details, and information on complications and arrhythmia outcomes. Data were aggregated and analysed centrally to evaluate procedural safety, efficacy, and variability in management strategies. A total of 198 patients (age 70 years, 37% female, 42% paroxysmal AF) with prior LAAO were included. Ablation was performed a median of 271 days after LAAO. After ablation, 84% of patients were on oral anticoagulation (OAC), most commonly for 2–3 months. Bleeding complications on OAC occurred in 6.7% of patients at a median of 20 (13, 70) days after ablation. Stroke or TIA occurred in five patients (2.6% of cases) within the follow-up period with two occurrences (1% of cases) the day after ablation. There were two cases of new or worsened peri-device leak after ablation.

**Conclusion:**

In a large, international registry of patients undergoing catheter ablation for AF with pre-existing LAAO devices, ablation was feasible, and device related complications were rare. Despite higher bleeding risk in an LAAO population, 84% of patients with prior LAAO tolerated short-term OAC post-ablation with bleeding events at expected rates for this population.

## Graphical Abstract

**Graphical Abstract:**
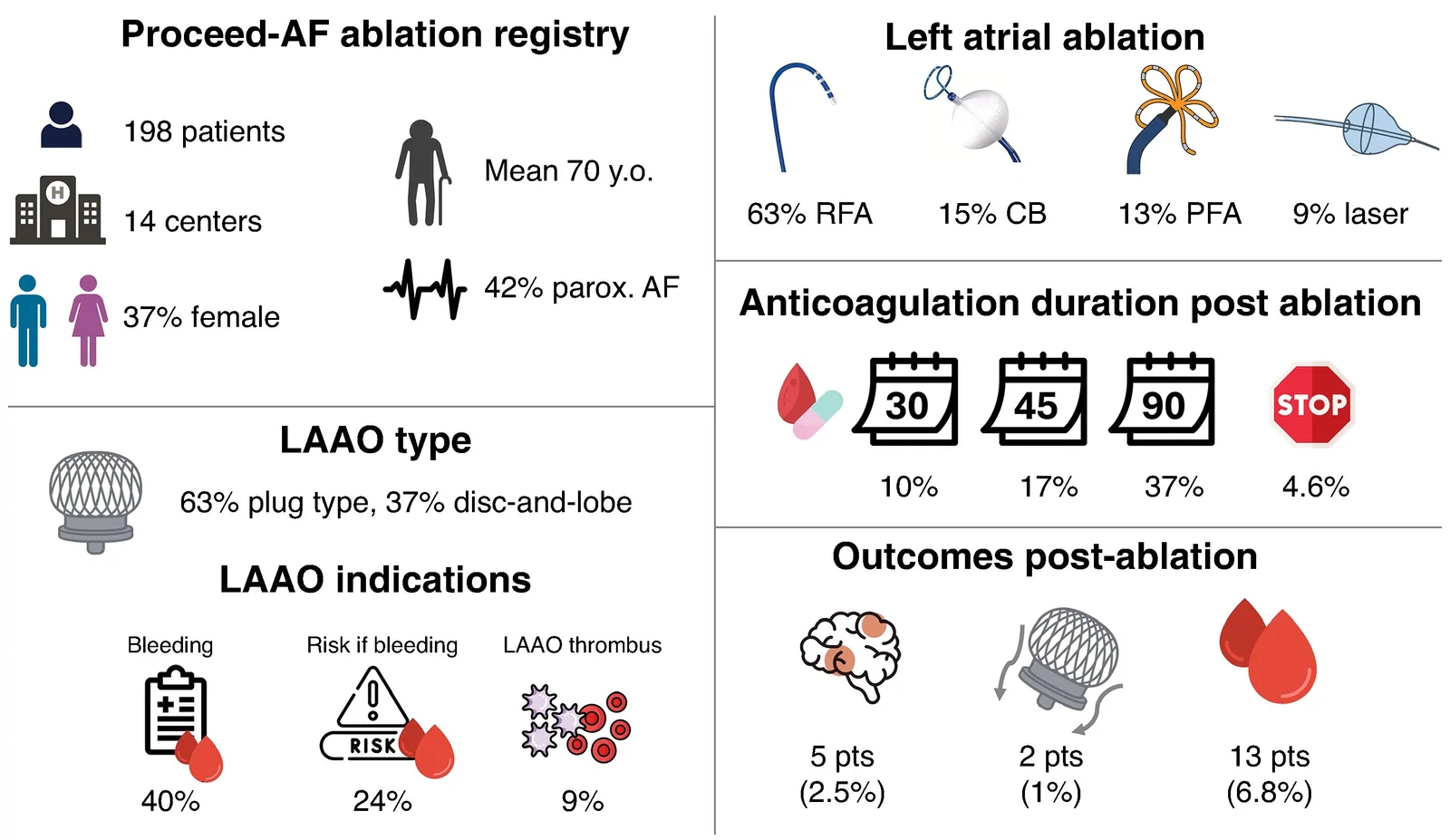

What’s new?

- Ablation of atrial fibrillation in patients with pre-existing left atrial appendage occlusion devices can be performed without a significantly increased risk
- Despite a population at high risk for bleeding, short-term oral anticoagulation in the peri-ablation period was well tolerated
- Novel complications such as worsening peri-device leaks were rare

## Introduction

Left atrial appendage occlusion (LAAO) is increasingly performed as an alternative to long-term oral anticoagulation (OAC) in patients with atrial fibrillation (AF). While it was initially utilized only in very high bleeding risk populations, with improvements in device technology and safety of the implant procedure, LAAO is now recommended to include patients at high bleeding risk but without a prior bleeding event, resulting in expansion of the eligible population for LAAO devices.^1^ Multiple recent and ongoing clinical trials have investigated the use of LAAO in patients who tolerate anticoagulation, and results from those studies may lead to a future where LAAO is available in patients at lower bleeding risk.^2,3^

Performing LAAO after catheter ablation for AF or even combining catheter ablation for AF with LAAO are increasingly common approaches, with recent randomized trial data demonstrating the safety and feasibility of this strategy.^4^ However, the OPTION trial enrolled patients at a relatively low risk for bleeding, and its protocol mandated completion of 3 months of anticoagulation following ablation, whether it was performed before LAAO placement or as a concomitant procedure. In contrast, limited evidence exists regarding the safety and effectiveness of performing catheter ablation for AF in patients with a pre-existing LAAO device. As the LAAO population grows and is increasingly offered to younger and healthier patients with lower bleeding risk, the demand for rhythm control strategies including ablation after LAAO is expected to rise. Consequently, it will become more common to perform catheter ablation procedures (potentially multiple times) in patients with prior LAAO.

Denying rhythm control strategies to patients with high bleeding risk who have undergone LAAO implantation leaves a vulnerable population without access to the most effective therapy for restoration of sinus rhythm, relief of symptoms, and improvement in quality of life. The objective of this real-world study was to investigate the safety and efficacy of catheter ablation for AF in high bleeding risk patients with a pre-existing LAAO device.

## Methods

### Study design and oversight

PROCEED-AF is an international multicentre registry evaluating ablation outcomes and anticoagulation usage patterns in patients with prior percutaneous LAA closure. Nineteen centres were invited to participate, of which 14 centres provided anonymized data to the co-ordinating site. Of the centres, eight are public academic medical centres and six are private academic centres. Six centres are located in Europe, and eight are in the USA. The study was approved by the institutional review board at the co-ordinating site (University of Chicago, Chicago, IL, USA) and individual centres provided data under data-sharing agreements between each institution and the co-ordinating site. The PROCEED-AF study is registered under ClinicalTrials.gov identifier NCT07580937.

Each site completed a common data collection sheet that contained predefined demographic, LAAO, ablation, antithrombotic, complication, and follow-up variables. A database manual with variable definitions accompanied the data sheet. Data were pooled at the co-ordinating centre for a one-stage central analysis. A study-specific investigator/centre variable was retained to track clustering by site. Submitted data were reviewed centrally for internal consistency, and date-derived intervals were recalculated from the submitted dates when available.

### Study population

Patients were included if they had undergone implantation of an endocardial LAAO device prior to the catheter ablation procedure. Concomitant ablation and LAAO cases were excluded from the analysis. Collaborating sites were asked to provide a set of predefined variables (see Supplementary material online, *Table S1*) on patients meeting inclusion criteria within the past 10 years. A database manual containing essential variable definition was provided to each centre.

### Data collection and merging

Data were collected at each centre using a common data sheet. Details of the variables collected are provided in the Supplementary material online. For the antecedent LAAO procedure, sites reported the device type and size, peri-device leak at implant, any complication, and follow-up leak assessment. Indications for LAAO were collected as predefined categories as were complications related to LAAO placement. Similarly, there were standardized ablation efficacy and safety outcomes, antithrombotic management, and arrhythmia follow-up collected.

### Statistical analysis

The data for all patients were pooled at the co-ordinating site, and a one-stage analysis was conducted. Clustering of the patients was tracked through the creation of a study-specific variable. Continuous variables are presented as mean ± standard deviation (SD) or median with interquartile ranges (IQR). Mann–Whitney *U* test was applied for comparison of continuous variables and the Pearson’s *χ*^2^ test or Fisher’s exact test for comparison of categorical variables, as appropriate. A *P*-value of <0.05 was considered significant. All statistical analyses were performed using the Statistical Software ‘R’ (R Foundation for Statistical Computing, Vienna, Austria).

## Results

### Patient population

A total of 198 patients who underwent endocardial LAAO and subsequent catheter ablation were included in this study (*Table 1*). Sites contributed a median of seven patients each (IQR 5.25–12.25) with full listing in Supplementary material online, *Table S2*. Median age was 70.7 years (IQR 62.8–78.4) with 37% female. Mean CHA_2_DS_2_-VASc score was 3.4 ± 1.39 and mean HAS-BLED was 3.0 ± 1.1 and distributions of scores are shown in *Figure 1*. The majority (63%) had a plug-type LAAO device (Watchman, Boston Scientific, Marlborough, MA, USA) and 36% had a disc-and-lobe design LAAO device (Amulet, Abbott, Abbott Park, IL or LAmbre, Lifetech Co, Shenzhen, China). Indications for LAAO were mostly history of bleeding and/or elevated risk of future bleeding (*Figure* *2A* and *B*). Assessment for leaks was performed in 84% of cases at LAAO implantation, and of those, 88% had no leak at that time. Follow-up imaging for leak assessment was performed at 45 days in 96% of the cohort, and of those, 83% had no leak. Of the other 17% who had a peri-device leak, the average size was 2 mm (IQR 2.0–3.0). Of the peri-device leaks that were identified, 50% occurred in plug-type LAAO devices and 50% in disc-and-lobe LAAO devices.

**Figure 1:**
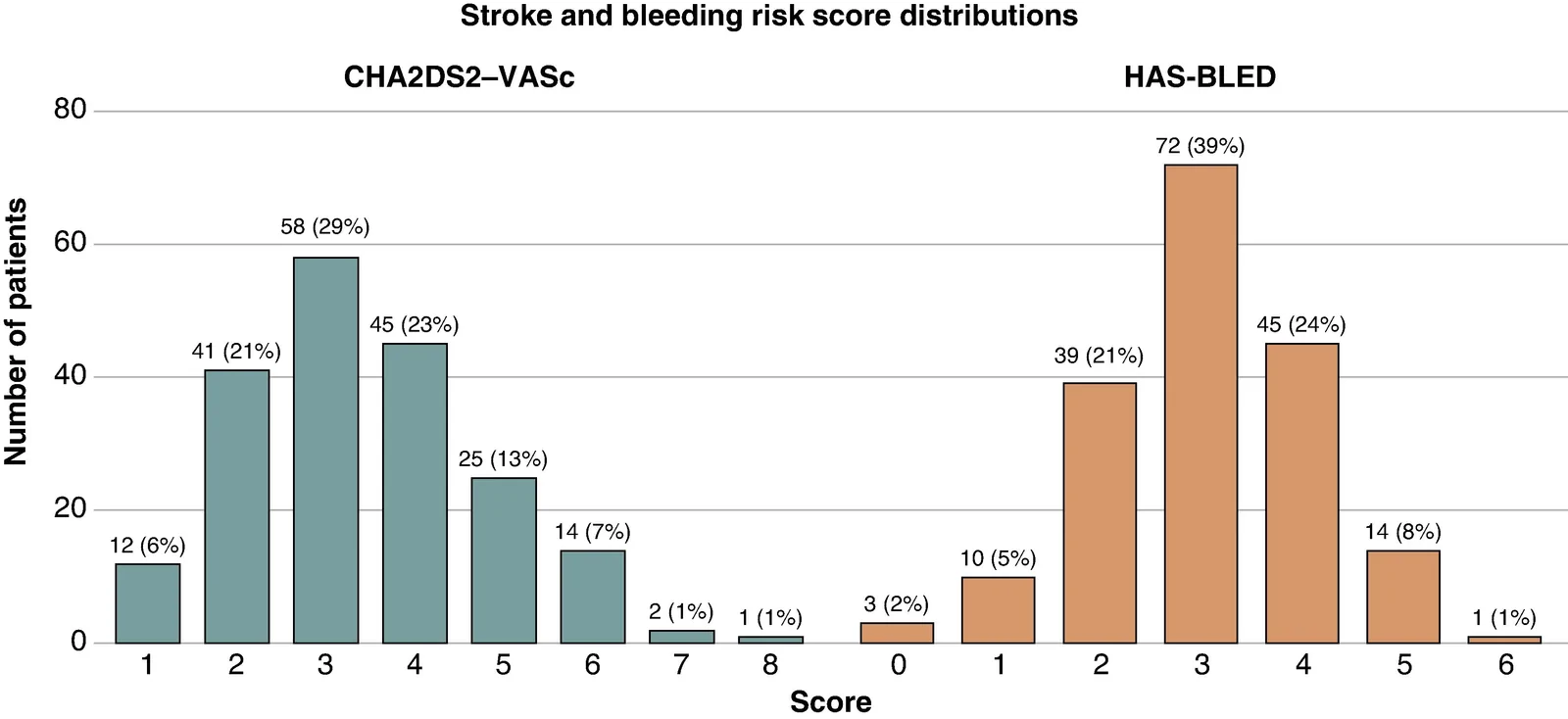
Distribution of CHA_2_DS_2_-VASc and HAS-BLED scores.

**Figure 2:**
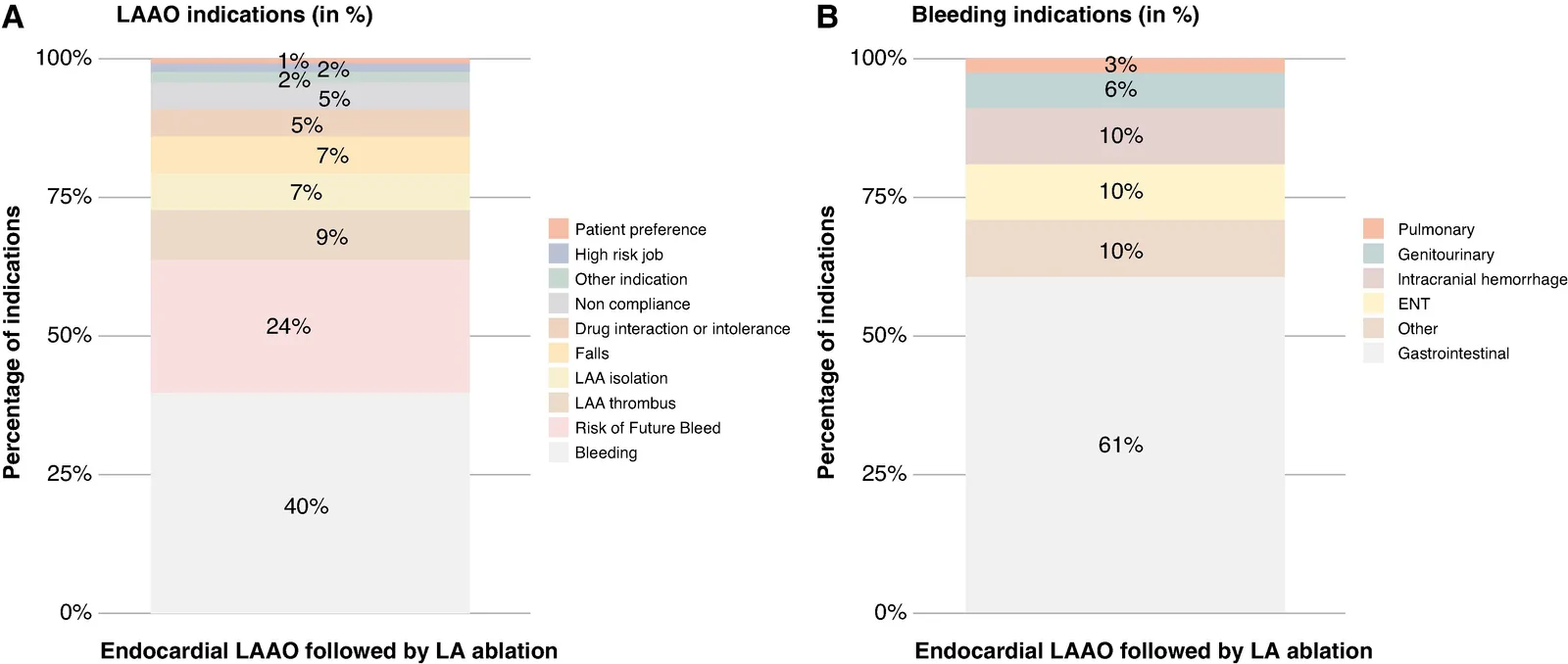
Overall indications for LAAO (panel *A*) and specific bleeding indications for LAAO (panel *B*). Indications for which patients had previously undergone LAAO and the per cent of patients who had met each of those indications. Specific types of bleeding for those patients who had undergone LAAO due to bleeding indication.

**Table 1:** Demographics of the study cohort

Characteristic	*n*	*n* = 198^a^
Age	198	70.7 (7.8)
Sex	198	
Female		74/198 (37%)
Male		124/198 (63%)
BMI (kg/m^2^)	169	29.8 (6.5)
EF (%)	182	55.8 (10.6)
LAVI (mL/m^2^)	85	44.1 (16.2)
LA diameter (cm)	143	30.1 (20.0)
Type of AF	198	
Paroxysmal		84/198 (42%)
Persistent		103/198 (52%)
Longstanding persistent		9/198 (4.5%)
Atrial tachycardia		2/198 (1.0%)
CHADS2VASc score	198	
1		12/198 (6.1%)
2		41/198 (21%)
3		58/198 (29%)
4		45/198 (23%)
5		25/198 (13%)
6		14/198 (7.1%)
7		2/198 (1.0%)
8		1/198 (0.5%)
CHADS2VASc score (continuous)	198	3.4 (1.4)
HAS-BLED score	184	
0		3/184 (1.6%)
1		10/184 (5.4%)
2		39/184 (21%)
3		72/184 (39%)
4		45/184 (24%)
5		14/184 (7.6%)
6		1/184 (0.5%)
HAS-BLED score (continuous)	184	3.0 (1.1)
History of tachycardia-induced cardiomyopathy	198	11/198 (5.6%)
History of CHF	198	54/198 (27%)
CIED	198	
None		179/198 (90%)
ICD		15/198 (7.6%)
PPM		4/198 (2.0%)
History of HTN	198	167/198 (84%)
History of diabetes	198	48/198 (24%)
History of stroke or TIA	198	35/198 (18%)
History of CAD	198	56/198 (28%)
History of PVD	198	17/198 (8.6%)
History of thrombocytopenia	198	8/198 (4.0%)
History of liver disease	198	7/198 (3.5%)
History of renal disease	198	33/198 (17%)
History of GI disease	198	57/198 (29%)
Indications LAAO		
Bleeding	198	79/198 (40%)
High risk of future bleed	198	47/198 (24%)
Falls	198	13/198 (6.6%)
High risk job	198	3/198 (1.5%)
Medication non-compliance	198	9/198 (4.5%)
Drug interaction or intolerance	198	10/198 (5.1%)
LAA isolation	198	13/198 (6.6%)
LAA thrombus	198	18/198 (9.1%)
Patient preference	198	2/198 (1.0%)
Other indication	198	4/198 (2.0%)
Type of LAAO	198	
Watchman		127/198 (64%)
Amulet		38/198 (19%)
LAmbre		33/198 (17%)
Size of interventional LAAO	196	26.0 (4.0)
Peridevice leak present at implant	198	
No leak		147/198 (74%)
Leak		20/198 (10%)
Not checked		31/198 (16%)
Median size of peridevice leak at implant (mm)	167	0.0 (0.0, 0.0)
Peridevice leak present at 45 days TEE follow-up	181	
No leak		142/181 (78%)
Leak		32/181 (18%)
Not checked		7/181 (3.9%)
Median size of peridevice leak at 45 days TEE follow-up (mm)	35	2.0 (2.0, 3.5)
Type of anticoagulation post-LAAO	195	
None		7/195 (3.6%)
Anticoagulant and antiplatelet		94/195 (48%)
DAPT		65/195 (33%)
Antiplatelet alone		27/195 (14%)
Anticoagulant alone		2/195 (1.0%)
Duration of anticoagulation post-LAAO	194	
None		8/194 (4.1%)
1–14 days		0/194 (0%)
15–30 days		8/194 (4.1%)
31–45 days		93/194 (48%)
46 days to 6 months		62/194 (32%)
>12 months		23/194 (12%)
Bleeding on anticoagulation post-LAAO	196	13/196 (6.7%)

^a^Median (p25, p75), mean (SD) or frequency (%).

### Ablation procedure

Catheter ablation was performed a median of 271 days (IQR 103–644) after LAAO. Ablation was performed for paroxysmal AF in 42% of the patients, and for persistent AF in 52%. Sixty-eight per cent of patients had a trans-oesophageal echocardiogram (TEE) performed immediately prior to the ablation procedure. Details of the ablation procedure are shown in *Table 2*. Radiofrequency ablation was used in 63% of cases, cryoballoon ablation was performed in 15%, laser balloon in 9.1%, and pulsed-field ablation (PFA) in 13%. All PFA cases utilized the pentaspline catheter (Farapulse, Boston Scientific, Marlborough, MA, USA). Eighteen per cent of patients had recurrent AF at a median of 219 days (IQR 72–579) post-ablation. Freedom from recurrent AF for both paroxysmal and persistent AF patients is shown in *Figure 3*. Procedure-related adverse events occurred in three patients (1.5%): One related to vascular access, one stroke and one TIA that both occurred the day following the ablation.

**Figure 3:**
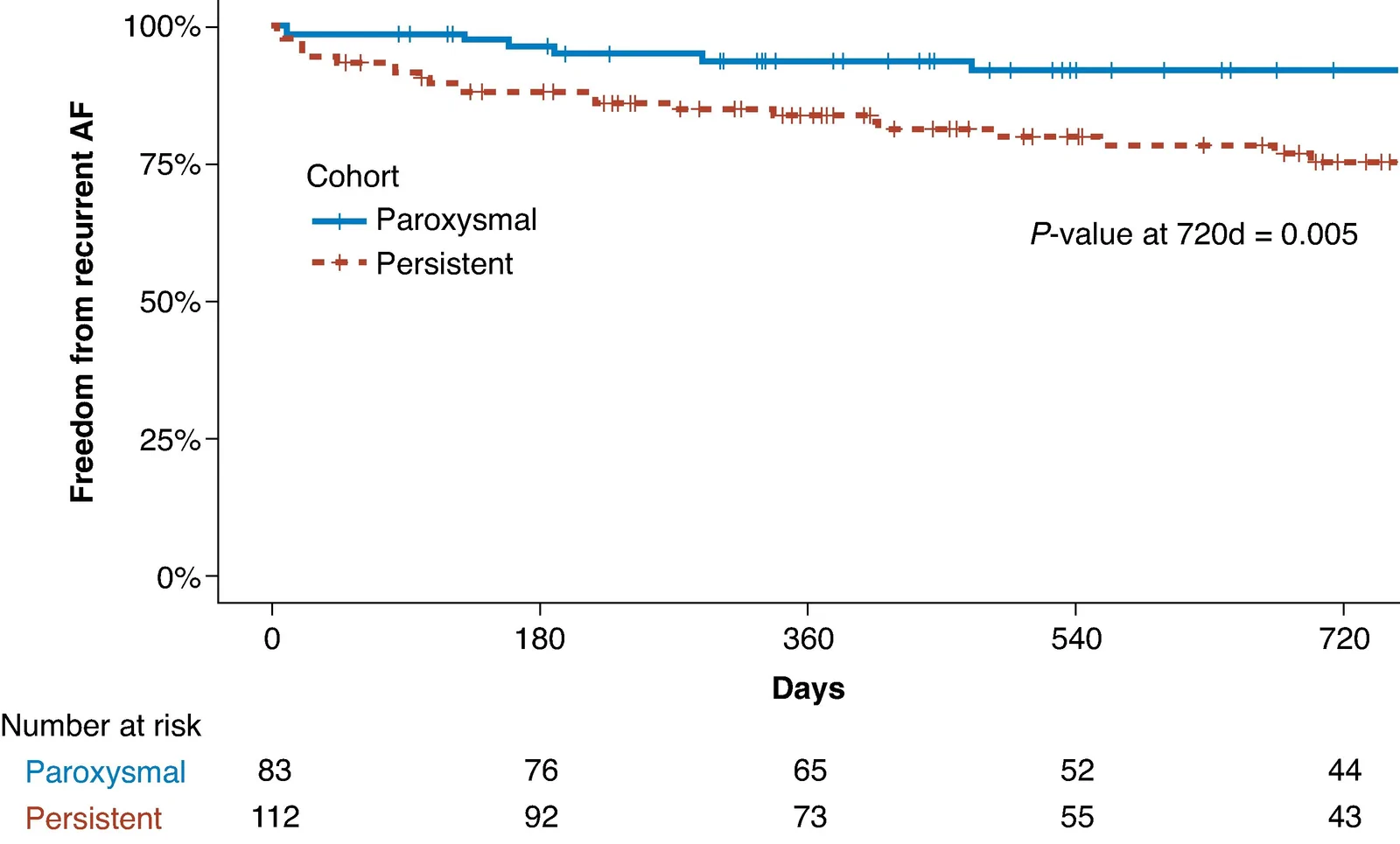
Kaplan–Meier curves for freedom from AF recurrence. Higher freedom from recurrent AF among patients who underwent ablation for paroxysmal AF as compared to persistent AF.

**Table 2:** Catheter ablation procedure characteristics

Characteristic	*n*	*n* = 194^a^
LA ablation modality	198	
Cryo		29/198 (15%)
RF		125/198 (63%)
Laser		18/198 (9.1%)
PFA		26/198 (13%)
Indication for LA ablation	191	
Drug resistant		178/191 (93%)
Tachycardia induced cardiomyopathy		8/191 (4.2%)
CHF exacerbations		5/191 (2.6%)
TEE prior to LA ablation	198	135/198 (68%)
Median days between LAAO and LA ablation	198	271 (103, 644)
LA ablation procedural time (min)	188	118.2 (67.6)
LA ablation fluoroscopic time (min)	182	10.7 (8.3)
Non PVI lesions		
CTI	198	59/198 (30%)
Posterior wall isolation	198	61/198 (31%)
Roof line	198	31/198 (16%)
Mitral line	198	24/198 (12%)
Vein of Marshall ablation	198	4/198 (2.0%)
Cardioversion during procedure	198	74/198 (37%)

^a^Median (p25, p75), mean (SD) or Frequency (%).

### Anticoagulation management and bleeding events

Prior to the ablation, 65% of the patients were on OAC for at least 3 weeks. After the ablation, 84.3% of patients were on OAC, and another 5.6% were on low molecular weight heparin. The most common durations of OAC after ablation were 60 days or 90 days (*Figure 4*). Bleeding complications occurred in 13 patients (6.7%) during the period of OAC following catheter ablation (*Table 3*). Bleeding events occurred at a median of 20 days post-ablation (IQR 13–70). OAC or antiplatelet therapy was modified (either reduced or stopped) in half of the bleeding cases.

**Figure 4:**
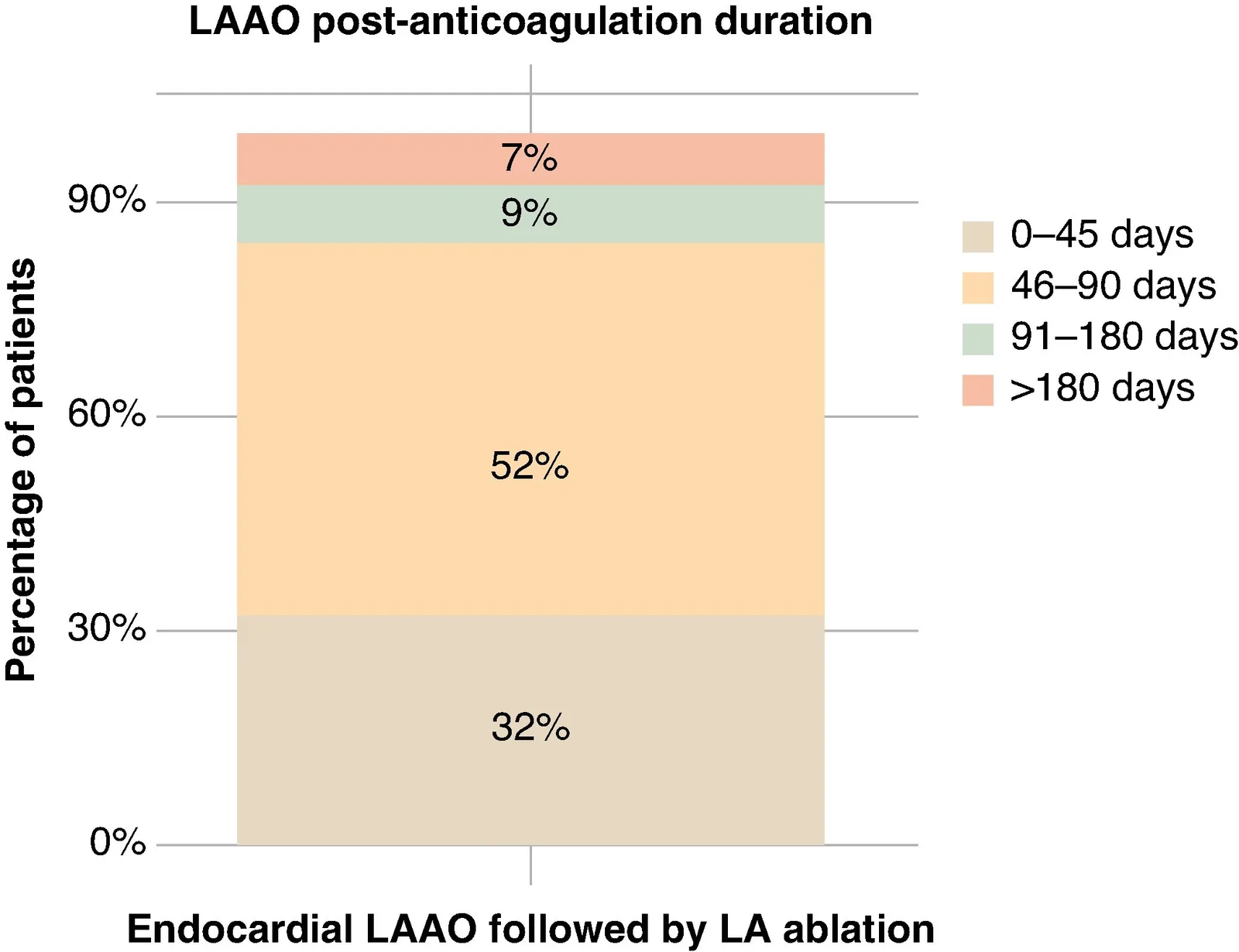
Post-ablation anticoagulation regimens. Duration of anticoagulation after catheter ablation with per cent of patients in each duration category.

**Table 3:** Bleeding complications during post-ablation anticoagulation

Type of bleeding	Days to bleeding	Type of anticoagulation/thrombotic regimen post-ablation	Number of days patient stayed on anticoagulant/thrombotic regimen	Anticoagulation/thrombotic regimen stopped sooner than planned	Type of anticoagulation/thrombotic regimen pre ablation
Tongue bleeding	15	Rivaroxaban	90	No	Rivaroxaban
Gastrointestinal	211	Rivaroxaban	Continued	No	Rivaroxaban
Gastrointestinal	15	Apixaban	60	No	Apixaban
Hematuria	58	DAPT	60	Antiplatelet stopped	DAPT
Gastrointestinal	13	Apixaban	30	OAC dose reduced	Aspirin or clopidogrel only
Hematuria	20	Apixaban	45	OAC dose reduced	DAPT
Hematuria	11	Apixaban	15	OAC stopped	Apixaban
Intracerebral haemorrhage	40	Rivaroxaban	60	No	Aspirin or clopidogrel only
Epistaxis	10	Apixaban	15	OAC stopped	DAPT
Gastrointestinal	113	Apixaban	60	No	Apixaban
Hemothorax	84	Apixaban	Continued	No	Apixaban
Hemoptysis	0	Apixaban	360	No	Apixaban
Hemarthrosis	70	Apixaban	180	OAC stopped	Apixaban

### Strokes and transient ischaemic attacks in follow-up

During and after the perioperative period, five patients experienced a stroke or transient ischaemic attack, with a median of 41 days post-ablation (IQR 1.0–51.0). Of these, two occurred the day following the ablation procedure. The other three occurred at 41, 51, and 372 days post-ablation. At the time of stroke, three patients were on anticoagulation (two on OAC, one on enoxaparin), one patient was on DAPT, and one was on aspirin alone (*Table 4*). The patient who had a stroke while on aspirin alone was also found to have a device-related thrombus during the work-up after the stroke. None of these events occurred in the small cohort of patients in whom PFA was used as the ablation energy type (*Figure 5*).

**Figure 5:**
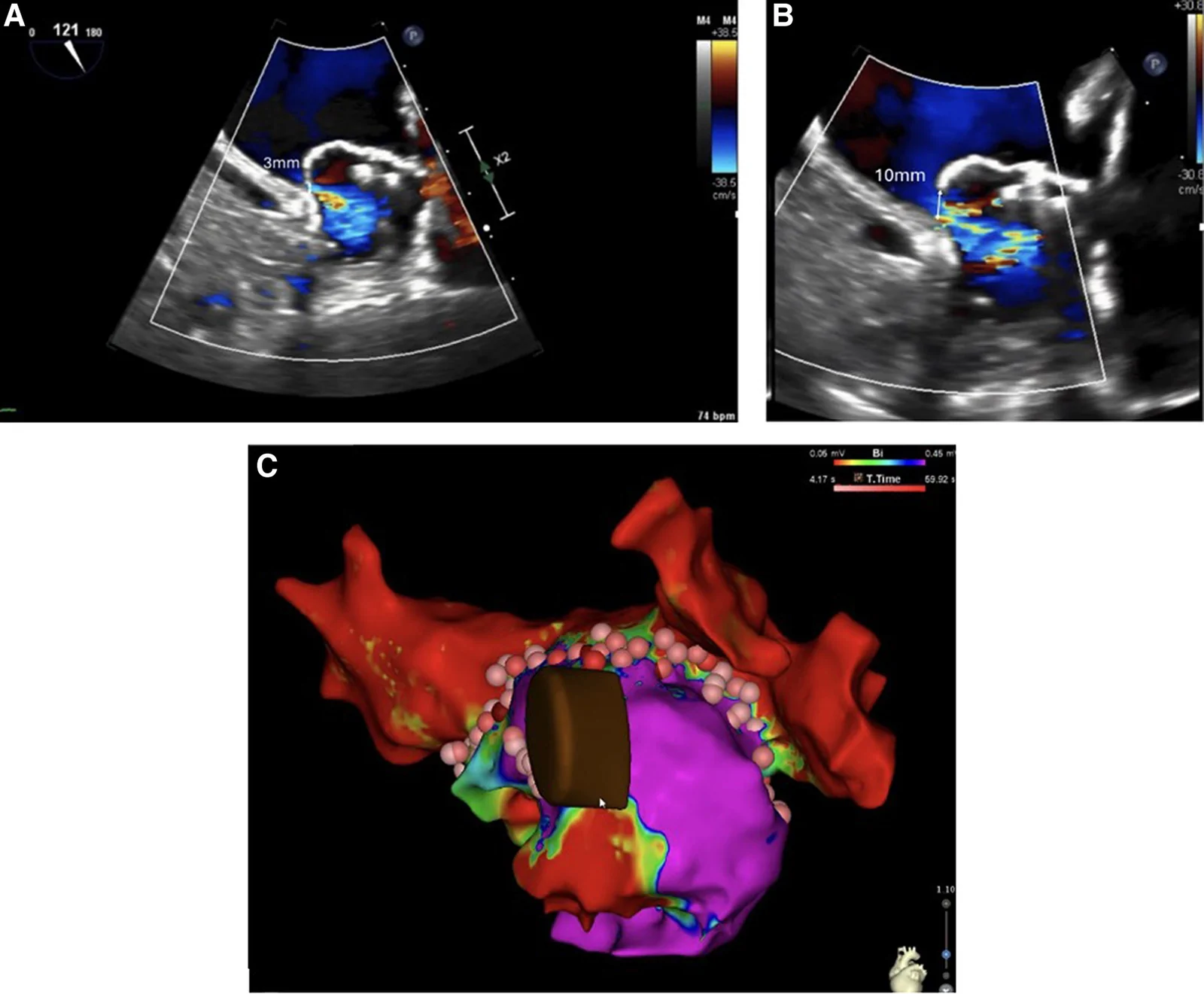
Trans-oesophageal images of small peri-device leak pre-ablation (panel *A*) that increased post-ablation (panel *B*). Left anterior oblique view of ablation lesions on 3D electroanatomic map with LAAO device portrayed in brown (panel *C*). Small peri-device leak prior to ablation (panel *A*) with similar image showing significantly larger peri-device leak after ablation (panel *B*). Anterior and Coumadin ridge ablation lesions shown on the 3D electroanatomic map in relation to the location of the LAAO device (shown in brown—panel *C*).

**Table 4:** Stroke and transient ischaemic attacks during follow-up

Stroke/TIA post-ablation	Number of days patient stayed on OAC/antiplatelet regimen	Type of OAC/antiplatelet regimen post-ablation	Number of days to stroke/TIA	Type of OAC/antiplatelet regimen at time of stroke/TIA	Ablation modality
TIA	90 days	DAPT	51 days	DAPT	Radiofrequency
Stroke	45 days	Apixaban	372 days	Aspirin	Laser
Stroke	60 days	Rivaroxaban	1 day	Rivaroxaban	Radiofrequency
TIA	Continued	Apixaban	1 day	Apixaban	Cryoballoon
Stroke	60 days	Enoxaparin	41 days	Enoxaparin	Radiofrequency

### Effects on existing left atrial appendage device

There were no cases of LAAO dislodgement during catheter ablation. There were two peri-device leaks that were new or worsened after catheter ablation. In the first case, there was no leak detected at implant, but a 3 mm leak was seen by TEE at 6 weeks post-implant. The catheter ablation procedure was performed 530 days after device implantation and was performed with radiofrequency ablation. The patient had another TEE 4 months after the ablation, and the leak was measured to be 10 mm at that time (*Figure 5*). In the second case, there was no leak detected at implant, nor at the 6-week assessment with TEE, or at an additional TEE performed 10 months after implant. The ablation was performed 352 days after the LAAO implant. A CT scan performed 1 month after the ablation did detect a small peri-device leak. The operator in that case noted difficulty performing ablation at the anterior and superior aspect of the left superior pulmonary vein, potentially due to the pre-existing LAAO device.

## Discussion

Catheter ablation in patients with pre-existing LAAO devices appears to be safe and effective, with outcomes comparable to those historically reported in patients without LAAO. These findings align with prior smaller studies, but this study represents the largest published cohort of this unique population to date.^5-8^ Traditionally, this high bleeding risk population undergoing LAAO has been relatively small but is expected to grow as LAAO becomes more common in younger and lower-risk patients who, by virtue of those characteristics, may be more likely to seek rhythm control strategies.

Because these patients cannot safely remain on long-term anticoagulation, they represent a uniquely vulnerable group in whom rhythm control strategies are often withheld. As their numbers expand, the demand for rhythm control, including catheter ablation, will inevitably increase, making it essential to define the safety and efficacy of these procedures in this setting.

An essential consideration in interpreting our findings is that this patient population underwent LAAO implantation mainly because of high HAS-BLED scores or partial/complete intolerance to long-term OAC. The indication for device placement in these cases was a strategy to mitigate stroke risk in patients for whom sustained anticoagulation was unsafe or unfeasible. This study cohort represented a high bleeding risk population, with a mean HAS-BLED score of 3. A HAS-BLED score of 3 or higher is the established threshold to define high bleeding risk in multiple international guidelines, including the ESC 2020 atrial fibrillation guidelines and the 2023 ACC/AHA/HRS AF guidelines.^9,10^ The HAS-BLED score has been extensively validated as a predictor of major bleeding in AF patients on OAC and thus provides a robust framework for risk stratification in this population.^11,12^ This context is critical, as it underscores why evaluating the safety of AF ablation in this population has clinical relevance.

In this study, catheter ablation included pulmonary vein isolation (PVI), as well as additional left atrial ablation in some cases at the discretion of the operator. Notably, there were no attempts to electrically isolate the occluded left atrial appendage. PVI was performed in all cases, without any novel complications due to the presence of an LAAO device. Rates of AF recurrence were consistent with past studies of AF ablation in patients with paroxysmal and persistent AF as well as a sub-study from the OPTION trial, which focused on patients who had undergone LAAO at the same time as ablation compared with after ablation.^13-16^ Our study now adds the cohort of patients who underwent ablation after LAAO with similar arrhythmia recurrence rates.

Ablation procedures in this study were primarily performed with thermal ablation—either radiofrequency or cryoballoon. As the field of catheter ablation moves rapidly towards PFA, attention is warranted regarding potential mechanical and electrical interactions between large PFA catheters and certain LAAO devices, particularly the disc-and-lobe design, which may impede successful isolation of superior pulmonary veins.^17-19^ In this cohort, over half of the patients had undergone LAAO with a plug-type device, while 36% had disc-and-lobe devices. As LAAO devices are increasingly implanted in younger patients, the need for AF ablation in the future may need to be considered with selection and/or positioning of the disc-and-lobe device. However, if a concomitant AF ablation and LAAO is pursued, PFA has been shown to be an efficient way to complete the procedure.^20^

A second key finding from this study was that OAC was commonly used for a short period after ablation, and bleeding complications during this time were rare. Although most patients underwent LAAO for the indication of bleeding (either by personal history of bleeding or determined to be high risk), they were able to tolerate OAC for the short term. The duration of time on OAC was variable, with most ranging from 30 to 90 days, and individual decisions about OAC duration may have been determined with consideration to that specific patient’s bleeding history, which is consistent with published strategies.^21^ Current treatment guidelines and expert consensus documents recommend a minimum of 2–3 months of uninterrupted anticoagulation post-ablation for AF.^1,9^ The rate of bleeding on OAC post-ablation in our study was comparable to that in a study of early post-operative bleeding after LAAO—when a similar population of patients was on their highest intensity OAC/antiplatelet therapy.^22^

In patients with prior LAA closure devices, the rationale for extended OAC following ablation differs from standard populations. The traditional concern of atrial mechanical stunning after cardioversion or sinus rhythm restoration is mitigated once the LAA has been excluded from systemic circulation, as the appendage is the predominant site of thrombus formation. A useful parallel comes from DCCV: Sharma *et al.* demonstrated that cardioversion in AF patients with well-seated LAAO devices and without post-procedure anticoagulation was free of thromboembolic complications in a cohort with similarly high-risk profiles.^23^ This analogy reinforces our findings that ablation following LAAO might also be conducted safely in selected high-risk patients without standard anticoagulation, provided there is appropriate imaging, surveillance, and a well-seated device, particularly if PFA is used.

Consequently, the need for prolonged anticoagulation in this group is less compelling. Moreover, preclinical and clinical data suggest that PFA induces less endothelial denudation and preserves vascular integrity to a greater extent than thermal energy sources, thereby potentially reducing procedure-related thrombogenicity. Taken together, these considerations support the safety of limited or no OAC following AF ablation in patients with well-healed LAA closure devices, provided that appropriate device surveillance and follow-up are maintained. In contrast, the scenario of concomitant LAA closure and catheter ablation represents a distinct setting in which acute device-related thrombogenicity remains a concern, thereby justifying short-term anticoagulation or antiplatelet therapy; this distinction highlights the importance of differentiating between patients undergoing ablation long after successful device implantation and those treated with a combined approach.

The decision to pursue AF ablation after the implantation of an LAAO device rather than before (or concomitantly) is often multifactorial and may depend on the specialty of the operator performing the LAAO. While most electrophysiologists might pursue AF ablation first for the benefits of restoring sinus rhythm, patients may also present to their clinics having already had LAAO.^24^ The recent OCEAN trial demonstrated that OAC can be stopped in patients who had undergone AF ablation at least a year earlier; the average CHA_2_DS_2_-VASc score in that trial was 2.2, which is notably lower than the average of 3.4 seen in the present study.^25^ In fact, the decision to pursue LAAO at all for many patients is one of the ongoing debates in light of recent trials. The CLOSURE-AF study, randomizing a high-risk population to LAAO or physician-guided care, found an increased risk of complications (primarily bleeding) with device implant that was driven by a surprisingly high rate of procedural bleeding.^26^ Meanwhile, in lower-risk patients, CHAMPION-AF demonstrated non-inferiority based on the prespecified statistical plan, but a numerically higher rate of strokes in the device arm, as compared to the OAC arm.^3^ However, as those patients were all able to tolerate OAC (by requirement for inclusion), the bleeding risks were not surprisingly low in the OAC arm. Taken alongside the OPTION trial, which evaluated OAC compared with device, either after ablation or concomitantly with ablation, these trials raise more questions about which patients should be having LAAO devices implanted.^27^ However, these devices are still being implanted, though perhaps with more thoughtful patient selection, and the issue of managing a subsequent ablation—whether an initial ablation or a redo procedure—will continue to occur.

There were two instances of worsened peri-device leak following catheter ablation in the present study. One leak was detected via CT post-ablation and was not present on prior TEE imaging. In that case, the operators noted difficulty targeting the anterior and superior portion of the left upper pulmonary vein. Although it is possible that the finding of a new leak was the result of a different imaging technique, catheter manipulation in that region may also have induced the new peri-device leak. The second case was a peri-device leak that increased from 3 to 10 mm, likely because of ablation near or on the existing LAAO device (*Figure 5*). While new or worsened leaks were rare post-ablation (1%), caution is warranted when ablating near an existing LAAO device. During endothelialization, the surface of LAAO devices eventually become covered by smooth muscle cells, which has been shown to be electrically active, making it difficult to distinguish them from the surrounding atrial tissue based on the presence of bipolar voltage values during electroanatomic mapping.^28^ Careful visualization, particularly with intracardiac echocardiography or fluoroscopy, may be needed to ensure ablation is performed on the Coumadin ridge and not over the actual device.^29^

Overall, the rate of peri-device leaks in this study is consistent with those reported in prior clinical trials.^4^ One study of isolating the LAA and occluding it did show increased leak risk, likely due to oedema in the LAA related to the ablation in that location.^8^ Another study of concomitant ablation and LAAO showed that ablation at the ostium of the left superior pulmonary vein using PFA as the energy source may cause oedema that can lead to more peri-device leaks in follow-up when the oedema resolves.^30^ Such acute effects of ablation with concomitant LAAO device implantation would not be expected to be seen in our study as ablation was performed subsequent to the LAAO device implant procedure.

The five cases of stroke or TIA in the study all occurred in patients who had undergone thermal or laser ablation. Two of the cases were peri-procedural, and the other three occurred several weeks (or even months) later. One late stroke patient was also found to have a device-related thrombus, which is a known risk even with current-generation LAAO devices and is a significant independent risk factor for stroke.^31,32^ While the stroke rate of 2.6% is higher than seen in recent trials of AF ablation, the mean CHA_2_DS_2_-VASc score in our study of 3.4 is also higher than in many of those ablation trials. For example, in AVANT-GUARD, the mean CHA_2_DS_2_-VASc score was 2.5, but there was still a 2% risk of stroke reported in the ablation arm.^25,33^ While surprising based on some literature, the population eligible for LAAO may truly have a higher stroke risk than the typical AF ablation population, as shown by CHAMPION-AF.^3^

This study is inherently limited by its retrospective, multicentre design. Although a common data collection instrument and variable dictionary were used at all 14 centres, the ablation technique, choice of energy source, intraprocedural monitoring, threshold for pursuing additional lesion sets, and utilization of loop recorders for post-ablation monitoring were determined by individual operators rather than a prospective protocol, and this heterogeneity cannot be removed retrospectively. Similarly, the stroke/TIA rate of 2.6% observed in this cohort exceeds that reported in several contemporary ablation trials; however, those trials enrolled populations with substantially lower baseline thromboembolic risk (mean CHA_2_DS_2_-VASc 2.2–2.5) than the present cohort (mean 3.4). We therefore cannot conclude from these data whether ablation itself confers additional stroke risk in patients with prior LAAO, only that patients with prior LAAO who undergo ablation experience thromboembolic event rates broadly consistent with their elevated baseline risk. A prospective, standardized study with systematic post-ablation imaging and rhythm monitoring, ideally with a non-ablated comparator group, would be required to resolve this question definitively.

The majority of ablations was performed with thermal ablation technologies though the field is moving rapidly towards PFA. Notably for patients who underwent PFA (11%) in this cohort, no stroke/TIA events were observed (*Figure 6*) though the PFA cohort was a small number and therefore larger studies are needed to truly assess any energy-specific risks. Although complications in ablation studies have been similar (or lower) between the energy sources, the findings of this study may not be generalizable to all combinations of LAAO and ablation energy. Given that the current study was not a prospective trial, the determination of a patient being ‘on’ anticoagulation was from a combination of being prescribed and from notes stating that the patient was compliant with anticoagulation medication. Also, the focus of the current study was to report the ablation outcomes in patients with a prior device and not to litigate the appropriateness of LAAO device implantation in patients referred for rhythm control. Further larger and prospective studies are required to convincingly ascertain the safety and efficacy of LAAO and subsequent catheter ablation.

**Figure 6:**
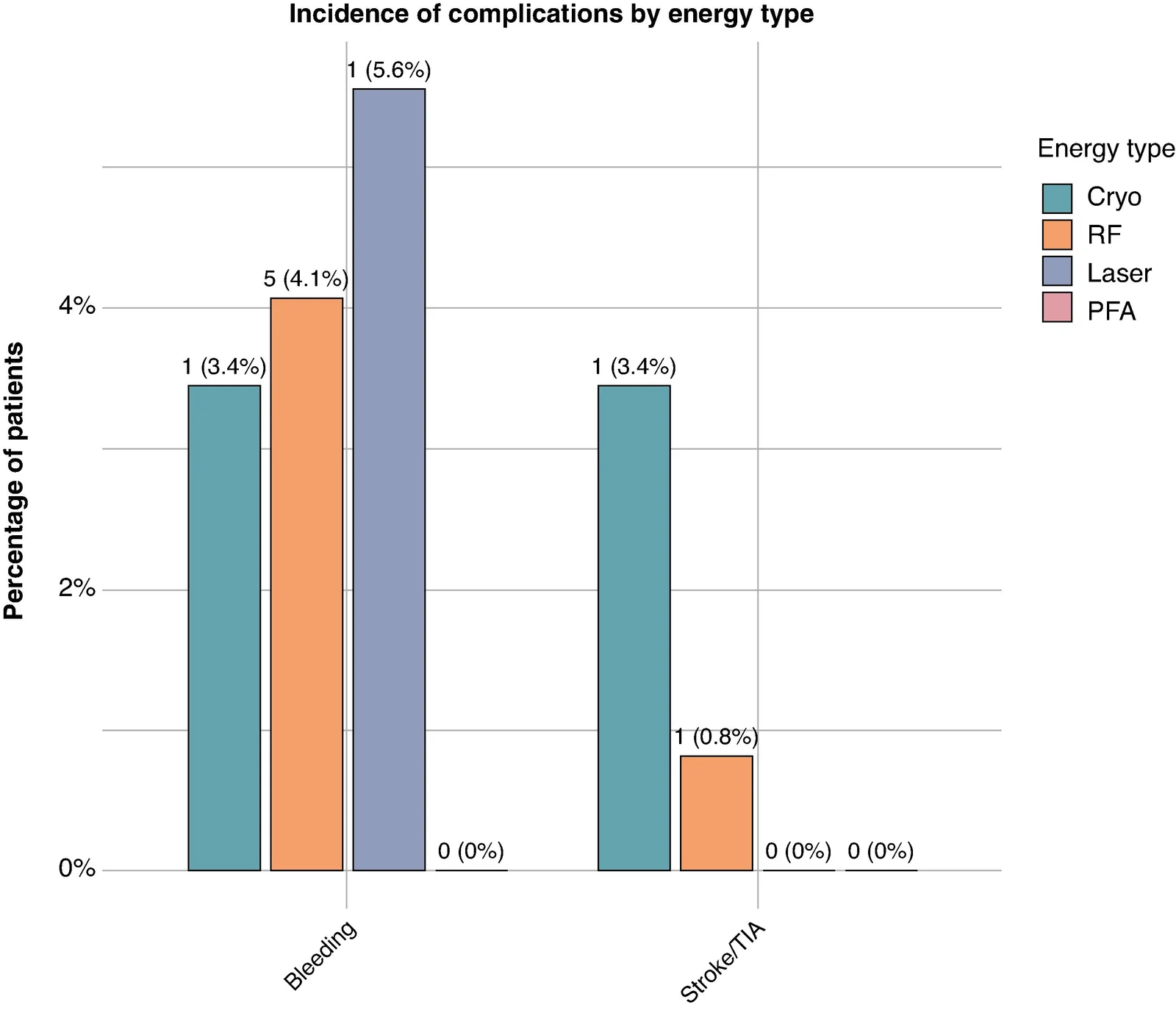
Procedural complications by energy type used. Complications within 30 days of ablation shown as distributed by the energy source used for the catheter ablation.

## Conclusions

In a large, international registry of patients undergoing catheter ablation for AF with pre-existing LAAO devices, ablation was feasible, and device-related complications were rare. Most operators utilized pre-ablation imaging of the LAAO device, and the majority of patients was treated with a 30–90 day period of short-term anticoagulation post-ablation with bleeding rates consistent with their bleeding risk.

## Perspectives

Competency in medical knowledge: Catheter ablation for AF has favourable outcomes in patients with pre-existing LAAO devices, and the vast majority of patients is able to tolerate short-term anticoagulation after the procedure.

## Supplementary Material

euag277_Supplementary_Data

## Data Availability

The data underlying this article are available in the article and in its online Supplementary material.
