# Supplementary material for: Safety and efficacy of left atrial ablation after percutaneous left atrial appendage occlusion in high-bleeding-risk patients with atrial fibrillation: a multicentre analysis from the PROCEED-AF ablation registry
Source: Europace. 2026 Sep 25;28(10):euag277. doi: 10.1093/europace/euag277 (PMC13688677; doi:10.1093/europace/euag277)
Supplement: Supplementary file 1 [file euag277_supplementary_data.zip › Supplement variables collected.docx]

# Supplement: Details of Variables Collected

Baseline variables collected before ablation included age, sex, body mass index, atrial fibrillation type, CHA2DS2-VASc score, HAS-BLED score, left ventricular ejection fraction, left atrial volume index, left atrial diameter, and cardiovascular and bleeding-risk comorbidities. The recorded comorbidities included heart failure, tachycardia-mediated cardiomyopathy, cardiac implantable electronic device, hypertension, diabetes, prior stroke or transient ischemic attack (TIA), coronary artery disease, peripheral vascular disease, thrombocytopenia, liver disease, renal disease, and gastrointestinal disease.

## LAAO Procedure and Device Variables

For the antecedent LAAO procedure, sites reported the date of implantation, device type, device size, indication for LAAO, peri-device leak at implant, follow-up leak assessment at approximately 45 days when performed, post-LAAO antithrombotic regimen and duration, bleeding on post-LAAO antithrombotic therapy, and LAAO-related complications. Peri-device leak was recorded in millimeters when a leak was identified and was also categorized centrally as no leak, leak, or not checked. LAAO-related complications were captured using predefined categories, including vascular complication, perforation, device dislodgement, device-related thrombus, and failure to implant.

Indications for LAAO were collected as predefined categories, including prior bleeding, high risk for future bleeding, falls, high-risk occupation, medication nonadherence, drug interaction or intolerance, prior LAA isolation, LAA thrombus despite anticoagulation, patient preference, and other indications. For descriptive tables that required a single indication per patient, overlapping indications were collapsed using a prespecified hierarchy that prioritized prior bleeding, falls, prior LAA isolation, LAA thrombus, high risk for future bleeding, medication nonadherence, high-risk occupation, drug interaction or intolerance, and patient preference; patients without any of these indicators were categorized as other.

## Ablation Procedure Variables

The ablation procedure was performed according to local operator practice. The registry did not mandate a uniform ablation catheter, mapping system, transseptal technique, intraprocedural anticoagulation protocol, target activated clotting time, or lesion delivery protocol. The procedural variables available for analysis were those specified in the common data collection sheet.

For each ablation, sites reported the ablation date, indication for ablation, ablation energy/modality, whether transesophageal echocardiography (TEE) was performed before ablation, pre-ablation antithrombotic regimen and duration, post-ablation antithrombotic regimen and planned duration, procedure time, fluoroscopy time, cardioversion during the procedure, cavotricuspid isthmus ablation, and additional non-pulmonary vein isolation lesion sets. Ablation modality was categorized as radiofrequency ablation, cryoballoon ablation, laser balloon ablation, or pulsed-field ablation. The core ablation procedure was pulmonary vein isolation. Additional lesion sets, when reported, were categorized as posterior wall isolation, roof line, mitral isthmus line, LAA isolation, vein of Marshall ablation, or other non-PVI ablation. Because the study was retrospective and procedure strategy was not protocolized, additional lesion sets reflected operator judgment and clinical indication at the treating center.

Pre-ablation TEE use was recorded as a binary procedural imaging variable. When performed, pre-ablation TEE was used according to local practice to evaluate the previously implanted LAAO device, peri-device seal, and/or left atrial thrombus before transseptal left atrial instrumentation. Post-ablation imaging was not mandated by the registry; therefore, post-ablation LAAO leak assessment reflects imaging obtained as part of local clinical care or event evaluation.

## Antithrombotic Management

Antithrombotic therapy was recorded before ablation and after ablation. Pre-ablation therapy was categorized by regimen and by duration, including whether anticoagulation had been administered for more than 3 weeks before the procedure. Post-ablation therapy was categorized as no antithrombotic therapy, single antiplatelet therapy, dual antiplatelet therapy, direct oral anticoagulant, vitamin K antagonist, low-molecular-weight heparin or other heparin therapy, or other agent. Direct oral anticoagulants were harmonized centrally from brand names to drug classes or generic names. Planned post-ablation therapy duration was recorded in days when available, with continued therapy recorded as a separate category. Early discontinuation or dose reduction of the post-ablation antithrombotic regimen was captured separately.

## Safety Outcomes

Safety outcomes were prespecified in the data collection sheet and included procedural complications of the index ablation, post-ablation bleeding while receiving antithrombotic therapy, post-ablation stroke or TIA, other thromboembolic events, device-related thrombus, LAAO device dislodgement, catheter entrapment, device perforation, pericardial effusion, vascular access complication or hematoma, and new or worsened peri-device leak after ablation. Index ablation complications were reported by each site using predefined categories. Stroke and TIA events were recorded separately from the general procedural complication field and included event date, MRI positivity when available, and whether clinical symptoms were consistent with stroke or TIA. Time to stroke/TIA was calculated from the ablation date to the event date.

Bleeding events after ablation were recorded when they occurred during the post-ablation antithrombotic treatment period. For each bleeding event, sites reported the event date, bleeding type, whether the patient was confirmed to be taking the prescribed antithrombotic regimen at the time of bleeding, and the intervention required. Bleeding interventions were categorized as no intervention, medication discontinuation, medication dose reduction, procedure, hospitalization, or transfusion when available. Time to bleeding was calculated from the ablation date to the bleeding date.

Device-related safety was assessed using the LAAO implant and follow-up leak measurements, the ablation complication field, and the specific post-ablation field for new or worsened peri-device leak. New or worsened peri-device leak after ablation was defined as a newly reported leak or an increase in a previously documented peri-device leak on imaging obtained after the ablation procedure. Because post-ablation device imaging was not systematically required, this endpoint should be interpreted as clinically detected new or worsened leak rather than an imaging-screened incidence estimate.

For descriptive energy-specific safety analyses, bleeding and stroke/TIA events were additionally summarized within 30 days after ablation by ablation modality. Events occurring after 30 days were retained in the overall follow-up safety analyses but were not counted as 30-day energy-specific events.

## Arrhythmia Follow-up and Effectiveness Outcome

Follow-up after ablation was conducted according to local clinical practice at each site. The registry captured last follow-up date and date of documented atrial fibrillation recurrence. The primary effectiveness endpoint for this descriptive analysis was freedom from documented atrial fibrillation recurrence during available follow-up. Time to recurrence was calculated from the ablation date to the first reported recurrence date. Patients without a reported recurrence were censored at the date of last follow-up. The registry did not mandate a uniform rhythm-monitoring protocol, use of implantable loop recorders, or a fixed blanking-period definition; therefore, recurrence reflects clinically documented recurrence as reported by participating centers.
