# Supplementary material for: Safety and efficacy of left atrial ablation after percutaneous left atrial appendage occlusion in high-bleeding-risk patients with atrial fibrillation: a multicentre analysis from the PROCEED-AF ablation registry
Source: Europace. 2026 Sep 25;28(10):euag277. doi: 10.1093/europace/euag277 (PMC13688677; doi:10.1093/europace/euag277)
Supplement: Supplementary file 1 [file euag277_supplementary_data.zip › Supplemental Table 2.docx]

Supplemental Table 2. Number of patients contributed by each center

| **Center number** | **Number of patients** |
| --- | --- |
| 1 | 7 |
| 2 | 1 |
| 3 | 6 |
| 4 | 7 |
| 5 | 36 |
| 6 | 7 |
| 7 | 5 |
| 8 | 17 |
| 9 | 3 |
| 10 | 14 |
| 11 | 5 |
| 12 | 74 |
| 13 | 7 |
| 14 | 7 |
