# Supplementary material for: Safety and efficacy of left atrial ablation after percutaneous left atrial appendage occlusion in high-bleeding-risk patients with atrial fibrillation: a multicentre analysis from the PROCEED-AF ablation registry
Source: Europace. 2026 Sep 25;28(10):euag277. doi: 10.1093/europace/euag277 (PMC13688677; doi:10.1093/europace/euag277)
Supplement: Supplementary file 1 [file euag277_supplementary_data.zip › Supplemental_Table 1.docx]

# **Supplemental Table 1. Data Dictionary: Variable Definitions and Timing**

This table lists the predefined variables, categories, and timing windows specified in the common PROCEED-AF data collection sheet and database manual used by all 14 participating centers. It is provided in response to reviewer requests for greater detail on how ablation, device, antithrombotic, safety, and effectiveness data were defined and captured across sites. As noted in the Methods and Limitations sections of the manuscript, these definitions were applied uniformly to the case-report form; they do not represent a prospectively mandated clinical protocol, and the choice of ablation technique, energy source, and follow-up imaging/monitoring remained at the discretion of each site's operators.

| **Domain** | **Variable** | **Predefined categories / definition** | **Timing** |
| --- | --- | --- | --- |
| Ablation | Ablation modality | Radiofrequency / Cryoballoon / Laser balloon / Pulsed-field ablation | Index ablation |
| Ablation | Non-PVI lesion sets | Posterior wall isolation / Roof line / Mitral isthmus line / LAA isolation / Vein of Marshall / Other | Index ablation |
| Ablation | Indication for ablation | Drug resistant / Tachycardia-induced cardiomyopathy / CHF exacerbations | Index ablation |
| Ablation | Pre-ablation TEE | Binary; performed to assess device seal/thrombus before transseptal access | Immediately pre-procedure |
| Ablation | Cardioversion during procedure | Binary | Index ablation |
| Device | Peri-device leak (implant) | Recorded in mm when present; centrally categorized as No leak / Leak / Not checked | At LAAO implant |
| Device | Peri-device leak (follow-up) | Recorded in mm when present; centrally categorized as No leak / Leak / Not checked | ~45 days post-implant |
| Device | New or worsened leak post-ablation | Newly reported leak, or an increase in a previously documented leak, on any imaging obtained after ablation | Post-ablation (non-mandated imaging) |
| Device | LAAO-related complications | Vascular complication / Perforation / Device dislodgement / Device-related thrombus / Failure to implant | At LAAO implant |
| Device | LAAO indication | Prior bleeding / High risk for future bleeding / Falls / High-risk occupation / Medication nonadherence / Drug interaction or intolerance / Prior LAA isolation / LAA thrombus / Patient preference / Other | Pre-LAAO |
| Device | Device type / size | Watchman / Amulet / LAmbre; size in mm | At LAAO implant |
| Antithrombotic | Pre-ablation regimen/duration | Regimen category; whether anticoagulation given >3 weeks pre-procedure | Pre-ablation |
| Antithrombotic | Post-ablation regimen | None / Single antiplatelet / DAPT / DOAC / VKA / LMWH or other heparin / Other agent | Post-ablation |
| Antithrombotic | Planned post-ablation duration | Days, or “Continued” if indefinite | Post-ablation |
| Antithrombotic | Regimen modification | Early discontinuation or dose reduction, captured separately from planned duration | Post-ablation |
| Safety | Procedural complication (index ablation) | Predefined categories (e.g., vascular access complication, pericardial effusion, etc.) | Index ablation |
| Safety | Stroke/TIA | Event date; MRI positivity when available; whether symptoms consistent with stroke/TIA; time-to-event from ablation date | Any point in follow-up |
| Safety | Bleeding event | Event date; bleeding type; confirmed adherence to prescribed regimen at time of event; intervention (none / medication discontinuation / dose reduction / procedure / hospitalization / transfusion); time-to-event from ablation date | During post-ablation antithrombotic period |
| Safety | 30-day energy-specific events | Bleeding and stroke/TIA events summarized separately within 30 days of ablation by ablation modality | Within 30 days of ablation |
| Effectiveness | AF recurrence | Date of first documented recurrence by clinically indicated monitoring (EKG/Holter, per local practice); censored at last follow-up if none reported | Post-ablation follow-up |
| Effectiveness | Last follow-up date | Date of most recent clinical contact | Post-ablation follow-up |
